# Faces under continuous flash suppression capture attention faster than objects, but without a face-evoked steady-state visual potential: Is curvilinearity responsible for the behavioral effect?

**DOI:** 10.1167/jov.20.6.14

**Published:** 2020-06-17

**Authors:** Andrew D. Engell, Henry M. Quillian

**Affiliations:** 1 Department of Neuroscience, Kenyon College, Gambier, OH, USA; 2 Department of Psychology, Kenyon College, Gambier, OH, USA

**Keywords:** faces, nonconscious processing, SSVEP, binocular rivalry, continuous flash suppression, curvilinearity

## Abstract

Face perception is a vital part of human social interactions. The social value of faces makes their efficient detection evolutionarily advantageous. It has been suggested that this might occur nonconsciously, but experimental results are equivocal thus far. Here, we probe nonconscious face perception using a novel combination of binocular rivalry with continuous flash suppression and steady-state visually evoked potentials. In the first two experiments, participants viewed either non-face objects, neutral faces (Study 1), or fearful faces (Study 2). Consistent with the hypothesis that faces are processed nonconsciously, we found that faces broke through suppression faster than objects. We did not, however, observe a concomitant face-selective steady-state visually evoked potential. Study 3 was run to reconcile this paradox. We hypothesized that the faster breakthrough time was due to a mid-level visual feature, curvilinearity, rather than high-level category membership, which would explain the behavioral difference without neural evidence of face-selective processing. We tested this hypothesis by presenting participants with four different groups of stimuli outside of conscious awareness: rectilinear objects (e.g., chessboard), curvilinear objects (e.g., dartboard), faces, and objects that were not dominantly curvilinear or rectilinear. We found that faces and curvilinear objects broke through suppression faster than objects and rectilinear objects. Moreover, there was no difference between faces and curvilinear objects. These results support our hypothesis that the observed behavioral advantage for faces is due to their curvilinearity, rather than category membership.

## Introduction

Faces are considered to be a special category of visual stimuli. In this view, the social and behavioral importance of these ubiquitous stimuli created an evolutionary pressure that resulted in sensory-cognitive processes and neural machinery specialized for face perception. But how special is special? The limited processing of the visual system necessarily means that some stimuli, particularly those outside of attentional focus and awareness, will only be processed superficially. Are faces equally vulnerable to this superficial treatment by the visual system, or does their evolutionary importance result in more complete processing, even when presented outside of awareness? The latter is an intuitively appealing notion, but empirical support has been equivocal (Axelrod, Bar, & Rees, 2015).

One approach to investigating nonconscious processing relies on the interocular suppression that occurs when each eye views a different image (binocular rivalry). Visual awareness will alternate between the stimuli, such that the initially suppressed image will reach awareness and vice versa. Continuous flash suppression (CFS) is a type of binocular rivalry paradigm that extends the potential duration of the suppression from seconds to minutes (Tsuchiya & Koch, 2005). Although CFS dramatically increases the duration of suppression, the suppressed images will eventually break through into awareness. Breakthrough of CFS (b-CFS) paradigms leverage this property by inferring differences in nonconscious processing if breakthrough times systematically vary across conditions (Jiang, Costello, & He, 2007).

This approach can be particularly powerful when paired with magnetoencephalography and electroencephalography, which can potentially yield an objective and temporally high-resolution electrophysiological marker of face-selective processing. However, this approach has yet to yield conclusive evidence, one way or the other, of selective nonconscious face processing. Several studies have reported an increased face-related response during nonconscious detection or discrimination of neutral faces, emotive faces, or inverted faces (Jiang & He, 2006; Jiang et al., 2009; Sterzer, Jalkanen, & Rees, 2009; Suzuki & Noguchi, 2013; Baroni et al., 2017), but several others have found no such evidence (Reiss & Hoffman, 2007; Harris, Wu, & Woldorff, 2011; Navajas, Ahmadi, & Quian Quiroga, 2013; Shafto & Pitts, 2015; Kume et al., 2016).

The inconsistent findings across studies can potentially be attributed to one or more methodological issues. In the current series of EEG and behavioral experiments, we investigate nonconscious face processing using a novel combination of methods in an effort to address the potential methodological limitations of prior work. Specifically, we record steady-state visually evoked potentials (SSVEP) while presenting faces and objects in a binocular rivalry paradigm using CFS. This approach addresses three possible limitations of prior studies.

First, it is possible that prior negative reports simply failed to detect a noisy, but nonetheless present, face-selective response. We address that issue by taking advantage of the fact that SSVEP has a high signal-to-noise ratio (SNR) relative to other EEG analysis techniques such as event-related potential (ERP) (Norcia, Appelbaum, Ales, Cottereau, & Rossion, 2015). Second, the inconsistent findings may be owed to the different blinding methods used across experiments. Axelrod, Bar, and Rees (2015) found that EEG studies that report no evidence of nonconscious face processing tend to use variations of masking paradigms, whereas those that find evidence tend to use variations of dichoptic stimulation (but see Izatt, Dubois, Faivre, & Koch, 2014; Shafto & Pitts, 2015). This finding might suggest a partial awareness during CFS (Mudrik, Gelbard-Sagiv, Faivre, & Koch, 2013; Gayet, Van der Stigchel, & Paffen, 2014; Stein & Sterzer, 2014) that results in false positives. The use of dichoptic stimulation in the current work can therefore be considered the more liberal approach, and thus potentially biased to find a positive result. However, this bias decreases the probability that a null result is a false negative. Third, the inconsistent findings may be owed to variation in what is considered “awareness” across studies (Faivre, Berthet, & Kouider, 2014; Peters & Lau, 2015)*.* As with the blinding differences noted elsewhere in this article, the concern is that differences in instructions and/or participant response biases (Rodríguez et al., 2012) could lead to false positives. That is, if participants use a conservative detection criterion, they might view images with some degree of conscious awareness without reporting it. The current work is less susceptible to such bias because SSVEP relies on the periodicity of the entire dataset and should, therefore, be less susceptible to individual differences in detection criterion. For example, a conservative detection criterion that results in a short window during which faces are consciously perceived but not reported would not result in a type I error (i.e., an SSVEP that seems to support nonconscious processing owing to the contribution of a brief period of conscious perception).

Here, we test the hypothesis that face processing occurs without the benefit of conscious awareness. In the first two studies, we used a novel combination of CFS and SSVEP to look for a face-selective response when faces were presented outside of awareness. Our predictions were two-fold: that faces would breakthrough CFS faster than non-faces, and that we would observe a face-selective SSVEP. Our results were inconsistent in that we observed the former, but not the latter. To reconcile this paradox, we report a third study in which we investigated whether a mid-level feature of faces—curvilinearity—was responsible for the faster breakthrough time, rather than high-level category membership, and thus the lack of face-selective neural signature.

## Methods

### Participants

Participants were recruited from the Kenyon College campus and surrounding community and compensated for their participation either monetarily or with research participation credit. All participants had normal or corrected-to-normal vision. All participants gave written and informed consent. The Kenyon College Institution Research Board approved this protocol. While piloting the experiment, we observed that some participants seemed to experience faster breakthrough if they had already participated in a different version of the experiment on a prior day. We therefore excluded from analysis any participants who had prior experience with the paradigm.

### Stimuli

**Figure 1.**
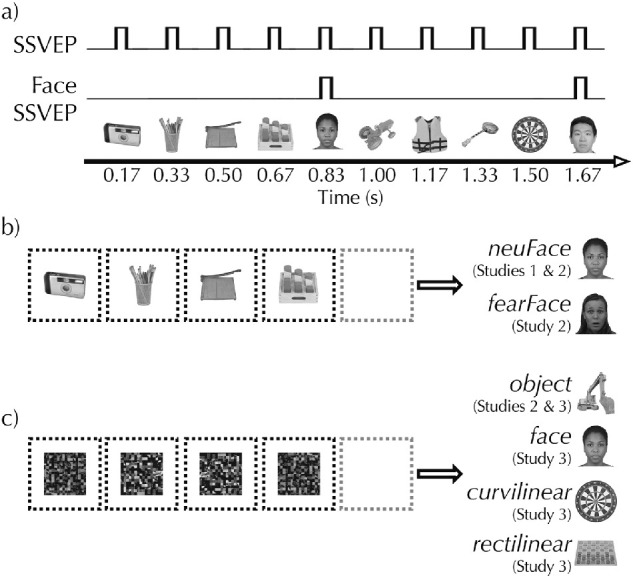
Stimuli and paradigm. A schematic example of the paradigm and hypothetical neural response (a). Stimuli are presented periodically every 167 ms. Most of these images are drawn from one category (e.g., objects), but every fifth stimulus is drawn for a different category (e.g., faces). Thus, image presentation (regardless of category) is fixed at 6 Hz, whereas the “oddball” stimuli (e.g., faces) are presented at one-fifth that rate, 1.2 Hz. Across the three studies, there were a total of six unique conditions. In Study 1, the *neuFace* condition (a) displayed common objects as the frequent stimuli and neutral faces as the oddballs. Study 2 included this same condition but added *fearFace* (b), in which faces displaying a fearful expression were the oddballs. Study 2 also included the *object* condition (c), in which scrambled images were displayed as the frequent stimuli and images of objects were the oddballs. Study 3 included four conditions that all used scrambled images as frequent stimuli (c). Each of the four conditions used images from different categories as the oddballs: objects in the *object* condition (as used in Study 2), neutral faces in the *face* condition, objects dominated by curvilinear edges in the *curvilinear* condition, and objects dominated by rectilinear edges in the *rectilinear* condition.

We converted all images to greyscale, 200 × 200 px jpegs with a resolution of 72 pixels per inch. We then matched the mean luminance across images (*M* = 135, *SD* = 45) with the SHINE MATLAB toolbox (Willenbockel et al., 2010) and added a cyan filter (00FFFF) using Photoshop CS6 (see Figure 1).

CFS stimuli consisted of a 20 × 20 matrix of 20 px^2^ cells. Every 166.67 ms the cell colors (red or white) would be re-randomized. This code was adapted from the code available at https://perso.univ-lyon2.fr/∼brogniar/notes/psychopy-continuous-flash/#head.flash_init.exp. A video demonstration of the paradigm is available at https://osf.io/ysx8e/.

### Experimental procedure

Stimulus presentation was controlled by PsychoPy (Peirce et al., 2019) and images were displayed on a 27” LCD display with a resolution of 1920 x 1080 and a refresh rate of 60 Hz. Participants were seated approximately 70 cm from the display; the exact distance varied to accommodate participant comfort. Interocular suppression was achieved using red-cyan anaglyph glasses. Participants wore the glasses throughout the entire experiment. In Studies 1 and 2, there were complementary conditions during which there was no flash suppression (*noCFS*). During the *noCFS* conditions participants wore the anaglyph glasses, but no flashing checkerboard was presented and thus all images were consciously perceived.

All *CFS* runs began with an instruction screen that directed participants to “Please focus on the cross in the middle of the screen for the entire experiment. If you see any images other than the flashing squares, please press any key.” The “flashing squares” were the CFS stimuli. The participant began the experiment with a button press, at which point a red fixation cross, centered within a black 500 × 500 px frame appeared at the center of the display. The frame and fixation cross remained onscreen throughout the presentation. The CFS began after 3 seconds, and the presentation of stimuli began 2 seconds after that. Images were presented at 400 × 400 px. The opacity of each of the superimposed images was set to 50%. However, the experimental images (e.g., faces and objects) were further decreased to 20% opacity in an effort to facilitate suppression.

The procedure and image opacity were the same in the *noCFS* runs, with two exceptions: 1) the flashing squares were not presented, and 2) the participant was instructed to simply focus on the cross in the middle of the screen for the duration of the run.

Each image remained on screen for 166.67 ms (10 frames at 16.67 ms per frame refresh), with an oddball stimulus presented as every fifth image. Thus, image presentation was at 6 Hz, whereas oddball presentation was at 1.2 Hz (Figure 1). One 833 ms “cycle” comprised four frequent stimuli and one oddball stimulus. This image presentation timing was modeled after several recent reports (see Norcia et al., 2015). Moreover, continuous flash has been found most effective at achieving suppression at frequencies of less than 10 Hz (Han, Lunghi, & Alais, 2016; Han et al., 2016; Zhan, Engelen, & de Gelder, 2019), particularly at or around 6 Hz (Zhu, Drewes, & Melcher, 2016; Zhan et al., 2019). Presentation would terminate after 50 cycles (41.67 s), or upon the participant indicating awareness of the suppressed images. In all three studies, there were three sequential runs of each *CFS* condition. The presentation order of the conditions was randomized across participants. The *noCFS* runs in Study 1 and Study 2 were always presented at the end of the experimental session after the participant had completed all of the *CFS* runs.

Note, the stimulus presentation rate and the flash rate were both 6 Hz. For Studies 1 and 2, this means that any SSVEP to frequent image presentation was confounded with any SSVEP response to the flash-suppression. We accepted this limitation because prior work (Han & Alais, 2018), and our pilot study observations, showed that suppression is most effective when the CFS frequency is matched to the stimulus presentation frequency. Critically, SSVEP to the oddball stimuli of interest is independent of any response to the CFS. The sole exception would be common harmonics of the oddball and CFS SSVEPs (e.g., the fifth harmonic of the oddball frequency is the same as the CFS fundamental frequency), but these were not included in analysis.

### Study 1

#### Participants

Data were collected from 30 participants. Four participants were excluded from this analysis. Two were excluded because they had previously seen a pilot version of the paradigm. One was excluded for failing to follow instructions and another owing to equipment failure. The remaining 26 participants included 11 males and 15 females with a median age of 21 years.

#### Stimuli and experimental procedure

In Study 1, objects were displayed as the frequent stimuli and neutral faces as the oddball stimuli. The two conditions differed as to whether these stimuli were presented with or without continuous flash suppression (*neuFace* and *neuFace_noCFS*, respectively). We selected 200 object images, excluding those that suggested animacy (e.g., dolls, toy animals) or any with a face-like appearance, from the set made available by Brady, Konkle, Alvarez, and Oliva (2008). We randomly selected 50 face images from the 74 faces available in the MR2 Face database (Strohminger et al., 2016). The *neuFace* condition was presented three times each (i.e., three runs), followed by two presentations of *neuFace_noCFS* condition.

### Study 2

#### Participants

Data were collected from 36 participants. Ten participants were excluded from this analysis. Four were excluded because they had previous experience with the paradigm. Three were excluded owing to equipment failure, two for failing to follow instructions, and one because of a metal plate in their skull. Finally, one was excluded because they experienced immediate breakthrough in all of the CFS conditions, indicating that the binocular rivalry was completely ineffective in achieving interocular suppression. The remaining 25 participants included 7 males and 18 females with a median age of 21 years.

#### Stimuli and experimental procedure

Study 2 was composed of six conditions: *neuFace*, *fearFace*, and *object*, each presented either with or without CFS (e.g., “*neuFace_noCFS*”). The *neuFace* condition was identical to the *neuFace* condition of Study 1. The *fearFace* condition displayed objects as the frequent stimuli and fearful faces as the oddball stimuli. Fearful faces oriented directly forward were taken from The Karolinska Directed Emotional Faces (Lundqvist, Flykt, & Öhman, 1998) stimulus set. In the *object* condition, grid scrambled objects were displayed as the frequent stimuli and objects as the oddball stimuli. Images were scrambled in MATLAB by dividing the image into a 20 × 20 matrix and then randomly shuffling the location of each cell in the matrix. The *neuFace, fearFace,* and *object* conditions were presented three times each (i.e., three runs), followed by two presentations of each *noCFS* condition.

### Study 3

#### Participants

Data were collected from 41 participants. Six participants were excluded for disregarding or misunderstanding instructions. The remaining 35 participants included 12 males and 23 females with a median age of 21 years.

#### Stimuli and experimental procedure

Study 3 was composed of four conditions: *face*, *object*, *curvilinear*, and *rectilinear*. In all conditions, scrambled objects were displayed as the frequent stimuli. In the *fa*ce condition, oddball images were seven neutral face images from the MR2 set described elsewhere in this article. In the *object* condition, oddball images were seven common objects (e.g., backhoe) that were selected for not being dominantly curvilinear or rectilinear. In the *curvilinear* condition, oddball images were seven common curvilinear objects (e.g., dartboard). In the *rectilinear* condition, oddball images were seven common rectilinear objects (e.g., chessboard). Each condition was presented three times (i.e., three runs).

### EEG acquisition and preprocessing

Continuous biopotential signals were recorded using the ActiveTwo BioSemi amplifier system (BioSemi, Amsterdam, the Netherlands). EEG was acquired from 64 scalp electrodes arranged in the 10/20 system. Two external electrodes were placed on the mastoids to be used as an offline reference. Two external electrodes were placed approximately 1 cm lateral and 1 cm inferior to the outer canthus of the left eye to record the horizontal and vertical electrooculogram, respectively.

All signals were digitized and recorded on an Apple Mac Mini running ActiView software (BioSemi) at a sampling rate of 2048 Hz. Off-line preprocessing and analysis were conducted with the EEGLAB (Swartz Center for Computational Neuroscience, La Jolla, CA, USA), and LETSWAVE6 (https://www.letswave.org/) MATLAB toolboxes, respectively.

Data were imported into EEGLAB, downsampled to 256 Hz, and bandpass filtered with a fourth-order Butterworth filter with cutoffs of 0.01 to 100 Hz. Data were then cropped to only include the 50 cycles (41.67 s) of stimulation plus an additional 1 s window before and after. For each run, the PREP pipeline (Bigdely-Shamlo, Mullen, Kothe, Su, & Robbins, 2015) was used to identify and interpolate bad channels and establish a “true” average reference. Runs in which more than 10 channels required interpolation were excluded from subsequent analysis. In Study 1, 24% and 20% of runs were excluded from the *CFS* and *noCFS* conditions, respectively. In Study 2, the range of excluded runs across all six conditions was 10.64% to 18.31%.

### Analysis

#### Behavior

The breakthrough time during CFS was compared to the maximum run duration by subtracting the former from the latter. Therefore, a larger value indicates a faster breakthrough of interocular suppression. In Study 1, we evaluated whether the breakthrough time was greater than zero using a one-sided one-sample *t* test. In Studies 2 and 3, we evaluated whether the breakthrough time varied across conditions with one-way repeated-measures analysis of variance (ANOVA) and Bonferroni-corrected pairwise comparisons. The Greenhouse–Geisser correction was used to correct for any violations of sphericity. ANOVA results were explicated with one-way paired-samples *t* tests. These tests were followed by Bayesian paired-samples *t* tests.

#### Electroencephalogram

The preprocessed data were imported into LETSWAVE6 (https://www.letswave.org/) and segmented into epochs that included twelve full cycles (10 s), starting with the third image of the second cycle and ending with the second image of the twelfth cycle. For complete 50-cycle runs (e.g., those without flash suppression), this resulted in four 12-cycle epochs per run. For runs that were terminated early owing to CFS breakthrough, the maximum number of nonoverlapping 12-cycle epochs were extracted and the remainder discarded. The decision to discard remainder cycles was motivated by the need for sufficient frequency resolution. A 12-cycle epoch yields a frequency resolution of 0.1 Hz (f resolution = 1/duration = 1/10 = 0.1 Hz).

The 12-cycle epochs were averaged for each participant and condition. The maximum number of epochs was 12 for *CFS* conditions and eight for the *noCFS* conditions. In an effort to ensure that the SNR of the *CFS* condition was equal to, or greater than, the SNR of the *noCFS* condition, the number of epochs included in each participant's condition averages was determined by the maximum number of available epochs in the *CFS* condition. For example, if a participant completed two, one, and three cycles in the three *CFS* runs, then only the first six *noCFS* runs would be included in the analysis. All completed *CFS* cycles were included in the analysis to ensure maximum sensitivity to SSVEPs evoked by nonconscious detection of suppressed images. In some cases, this meant that more cycles were included in the *CFS* than the *noCFS* condition average.

After discarding participants with fewer than one full cycle (i.e., participants who experienced breakthrough within the first 10 seconds of all three *CFS* runs for a given condition) and runs with an excessive number of noisy channels, the following sample sizes and average number of epochs were available for SSVEP analysis. In Study 1: *neuFace* (*N* = 19), *CFS* = 7.5, *noCFS* = 6.1. In Study 2: *neuFace* (*N* = 20), *CFS* = 8.8, *noCFS* = 6.05; *fearFace* (*N* = 19), *CFS* = 9.4, *noCFS* = 6.7; *object* (*N* = 20), *CFS* = 11.1, *noCFS* = 7.5. Note, these Study 2 samples represent subsets of the same 22 participants, with 17 participants in common across all conditions.

A fast-Fourier transform was applied to an average of all available epochs for each participant and condition. The results were then baseline corrected by subtracting the surrounding 16 bins (eight bins on each side) excluding the local maximum and minimum. We chose eight bins on each side to avoid contribution from neighboring harmonics, which occurred at multiples of 1.2 Hz, or 12 bins with our frequency resolution of 0.1 Hz. To facilitate visualization, each bin was *z*-normalized relative to the same range of bins described above.

We visually inspected the scalp distribution of power at the face evoked frequency for the *neuFace-noCFS* conditions and found the largest response at electrodes over the right occipitotemporal scalp: P8, PO8, and P10 (see Figures 3 and 4). This result is consistent with several prior SSVEP studies of face perception (Ales, Farzin, Rossion, & Norcia, 2012; Boremanse, Norcia, & Rossion, 2013; Liu-Shuang, Norcia, & Rossion, 2014) and so these electrodes were selected as the region of interest for subsequent analysis. Results of analysis run on analogous electrodes of the left hemisphere (P7, PO7, and P9) are available in the Supplementary Materials. The statistical tests described below were run on the average of the first and second harmonics averaged across all the three region of interest sites. Averaging of the first and second harmonics was done to maximize SNR. In our pilot studies of SSVEP face presentation, we found that the second harmonic was consistently as large, or larger than the fundamental frequency (aka first harmonic). The amplitudes of the third and fourth harmonics, in contrast, were more variable.

We used Bayesian one-sample *t* tests (Jeffreys, 1961) as implemented in JASP 0.10.2 (JASP Team, 2019) to test whether the SSVEP was greater than zero in either the *neuFace_noCFS* or *neuFace* conditions (Study 1 and Study 2) or in the *fearFace_noCFS*, *fearFace* conditions, *object_noCFS,* or *object* conditions (Study 2). The null hypothesis for each condition states that the SSVEP is equal to zero, H_0_: δ = 0. The alternative hypothesis states that effects are positive values and thus δ was assigned a Cauchy prior distribution with r = 1 / √2, truncated to allow only positive effect sizes. We used one-sample *t* tests rather than paired-sample *t* tests or repeated-measures ANOVAs because we were interested in whether either condition evoked a significant response, not whether the magnitude of any such response varied as a function of CFS/noCFS condition. For example, a paired-samples *t* test might show that the SSVEP during conscious perception was greater than during nonconscious perception, but this would not tell us whether the latter evoked a response greater than zero.

In study 2, we also used Bayesian paired-sample *t* tests to compare the SSVEPs evoked by *neuFace* and *fearFace* and to compare the SSVEPs evoked by *neuFace_noCFS* and *fearFace_noCFS.* The *object* conditions used scrambled images as frequent stimuli, whereas the *neuFace* and *fearFace* conditions used objects as frequent stimuli. Direct comparisons with the *object* conditions would, therefore, be uninterpretable and so were not included in the analysis. Complementary frequentist one-sample one-sided *t* tests were also performed.

## Results

### Study 1: Behavioral results

**Figure 2.**
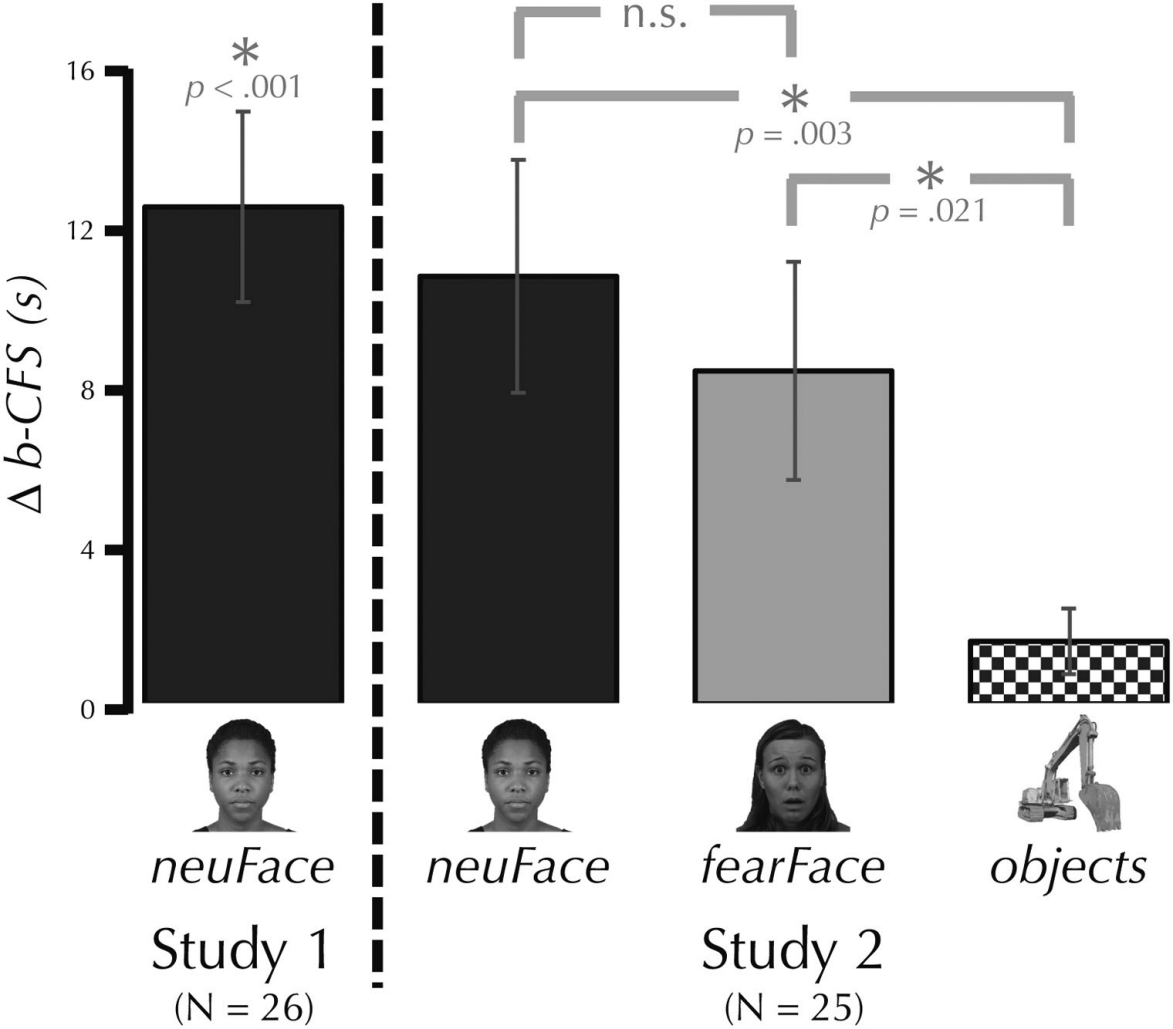
CFS breakthrough time (b-CFS) advantage in Studies 1 and 2. The bar graph shows the difference between the entire possible run duration and the actual average run duration, so a larger number indicates a faster breakthrough time. Note: the b-CFS results from both Study 1 and Study 2 are presented here, but each included an independent sample and was subject to a different analysis. The results of Study 1 (to the left of the vertical dashed line) were analyzed using a one-way one-sample *t* test. The results of Study 2 show the results of Bonferroni-corrected post hoc tests (see Methods).

Across all participants (*N* = 26) the breakthrough time during CFS was significantly faster than the full run duration, *M*_diff_ = 12.44 s, *SD* = 11.84 (Figure 2). A one-sample one-sided *t* test found that this was a significant difference, *t*(25) = 5.36, *p* < .001, *d* = 1.05. This finding was true even when we restricted the analysis to include only the subset of participants who were included in the SSVEP analysis, *M*_diff_ = 8.55 s, *SD* = 9.21; *t*(18) = 4.05, *p* < .001, *d* = 0.93.

### Study 1: EEG results

**Figure 3.**
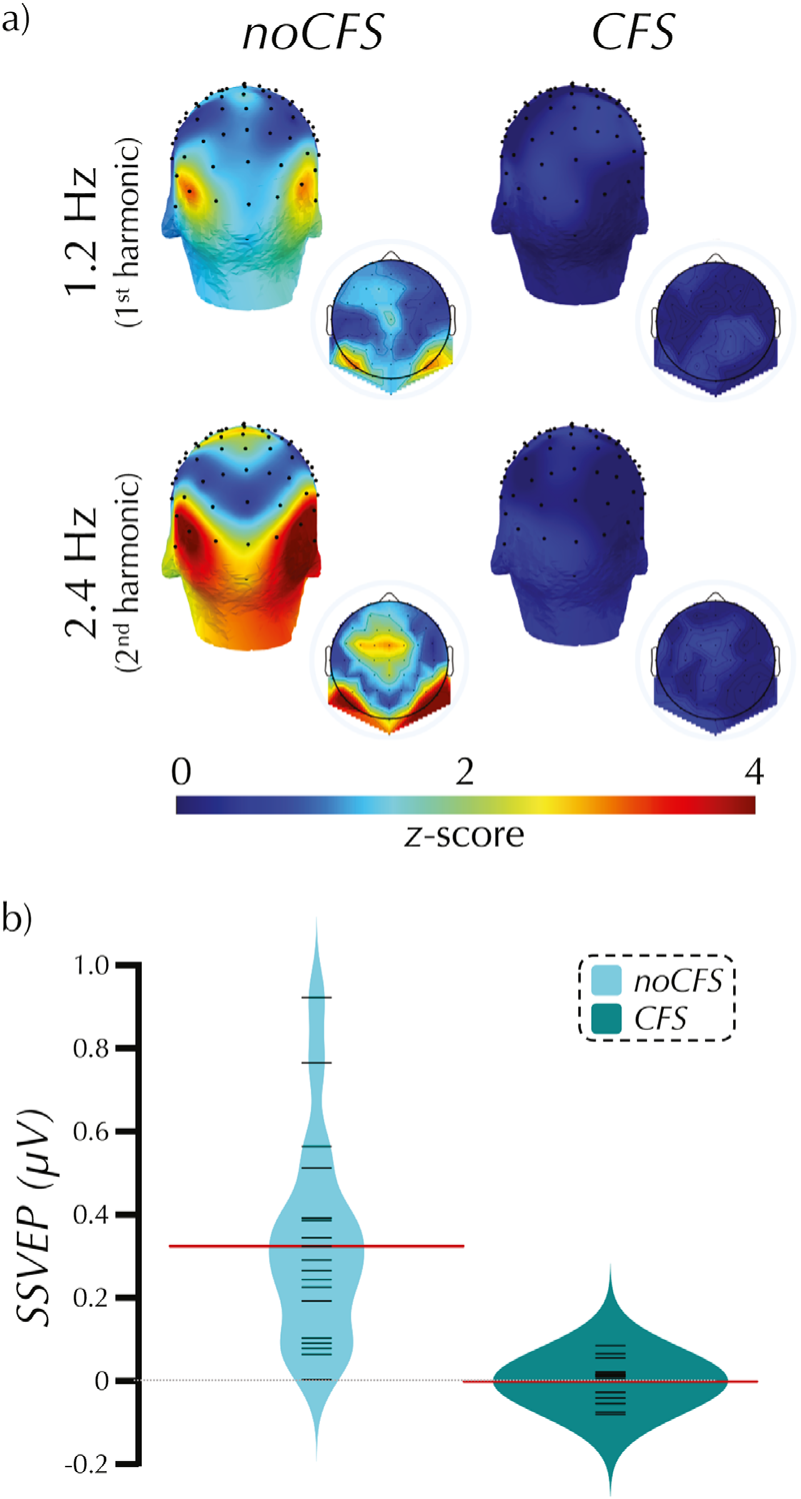
Study 1 SSVEP to neutral faces with and without CFS. These three-dimensional (larger) and two-dimensional (smaller inset) scalp maps display the distribution of normalized power at the first (aka fundamental) and second harmonics of the oddball presentation frequency (a). During *noCFS* there was no interocular suppression and the participants were therefore consciously aware of all presented stimuli. During *CFS* there was interocular suppression and the participants were therefore unaware of the stimuli of interest presented to the ‘suppressed’ eye. The bean plots (b) display the average amplitude of the response (µV) combined across the first and second harmonics and across three electrodes of interest: P8, P10, and PO8 for each participant. For each condition, the plot displays the individual participant results (black lines), the distribution density of the results (mirrored across the vertical axis), and the mean response (red line).

Figure 3 shows the scalp distribution of power at the first and second harmonic, and the average SSVEP to the *CFS* and *noCFS* conditions. For the *neuFace*_*noCFS* condition, *M* = 0.33 µV, *SD* = 0.24, we found extreme evidence, BF = 3736, that the observed data are more likely under H_1_, δ > 0, than under H_0_, δ = 0. In contrast, for the *neuFace* condition, *M* < 0.01 µV, *SD* = 0.05, we found moderate evidence, BF = 0.28, that the results are more likely (specifically, 3.56 times more likely) under H_0_, δ = 0, than under H_1_, δ > 0.

The results of the frequentist one-sample one-sided *t* tests were qualitatively the same as the Bayesian tests. A significant response was evoked by *neuFace_noCFS*, *t*(18) = 5.97, *p* < .001, *d* = 1.37, but not *neuFace* condition, *t*(18) = 0.22, *p* = .42. See Supplementary Material 1.1, Figure S1 for left hemisphere results.

### Study 2: Behavioral results

Across all participants (*N* = 25), a one-way repeated-measures ANOVA showed that the breakthrough times significantly varied as a function of condition, *F*(1.65, 39.7) = 9.66, *p* < .001 (Figure 2). Bonferroni corrected post hoc tests showed that *object* breakthrough time was significantly slower than for *neuFace*, *M*_diff_ = 8.88 s, SD = 12.00, *t*(24) = 3.70, *p* = .003, *d* = 0.74, and *fearFace*, *M*_diff_ = 6.58 s, *SD* = 11.16, *t*(24) = 2.95, *p* = .021, *d* = 0.59. Breakthrough times did not did not differ between *neuFace* and *fearFace*, *p* = .462*.* Consistent with this result, the Bayesian paired-samples *t*-test found anecdotal evidence, BF = 0.55, in support of the null hypothesis of no difference.

A second repeated-measures ANOVA was run on the subset of participants who were included in the SSVEP analysis. However, only 17 of the 22 participants contributed data to all conditions, and therefore the remaining five were held out of this analysis. As with the full sample, breakthrough times significantly varied as a function of condition, *F*(1.97, 31.57) = 4.50, *p* = .019. Bonferroni corrected post hoc tests showed that *object* breakthrough time was significantly slower than for *neuFace*, *M*_diff_ = 6.77 s, *SD* = 9.93, *t*(16) = –2.81, *p* = .038, *d* = 0.68, but not *fearFace*, *M*_diff_ = 4.32 s, *SD* = 9.31, *p* = .221. Breakthrough times did not differ between *neuFace* and *fearFace*, *p* = .838*.* Consistent with this result, the Bayesian paired-samples *t*-test found anecdotal evidence, BF = 0.41, in support of the null hypothesis of no difference.

### Study 2: EEG results

**Figure 4.**
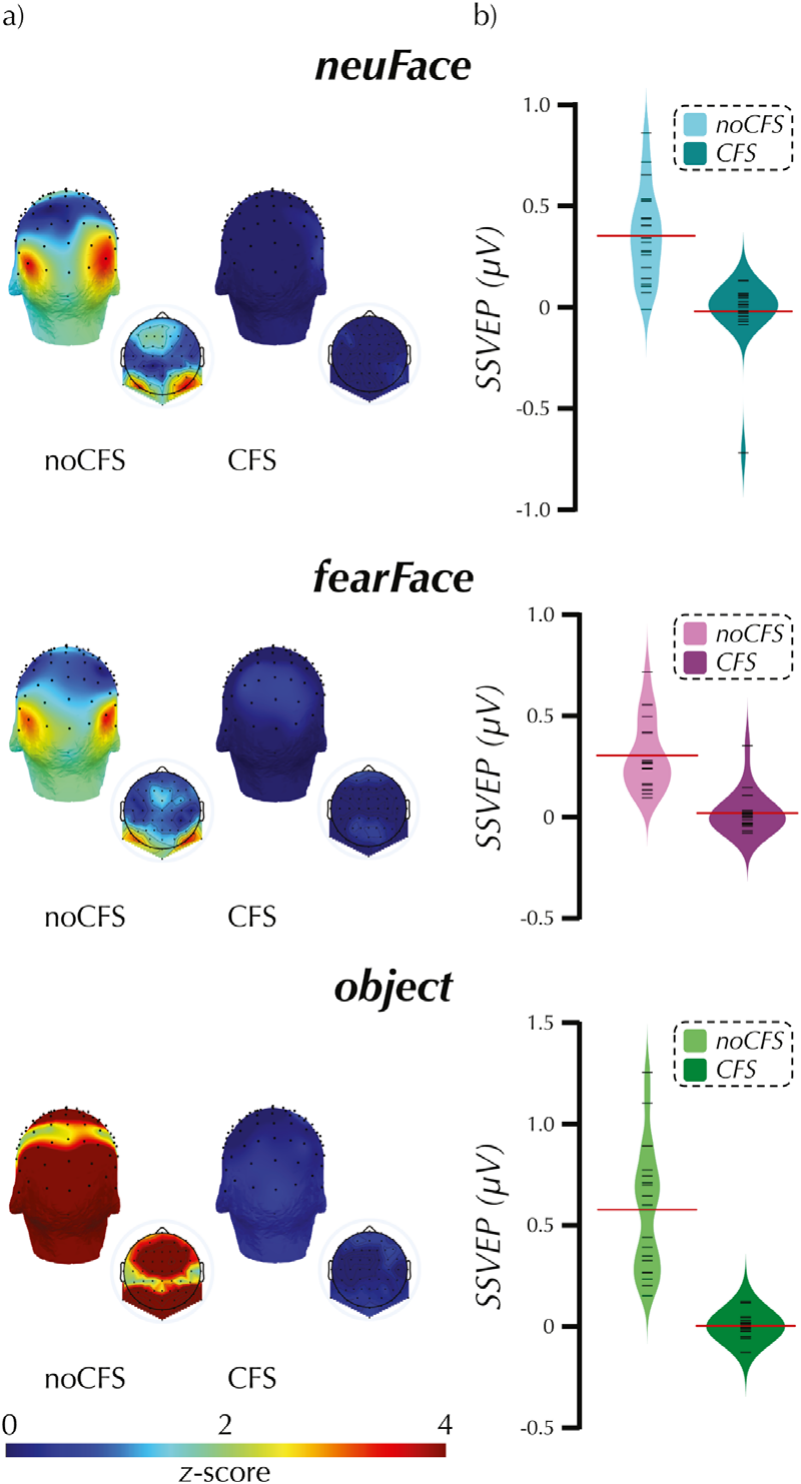
Study 2 SSVEP. These three-dimensional (larger) and two-dimensional (smaller inset) scalp maps display the distribution of normalized power at the first harmonic (aka fundamental) of the oddball presentation frequency (a). During *noCFS* there was no interocular suppression and the participants were therefore consciously aware of all presented stimuli. During *CFS* there was interocular suppression and the participants were therefore unaware of the stimuli of interest presented to the “suppressed” eye. The bean plots (b) display the average amplitude of the response (µV) combined across the first and second harmonics and across three electrodes of interest: P8, P10, and PO8 for each participant. For each condition, the plot displays the individual participant results (black lines), the distribution density of the results (mirrored across the vertical axis), and the mean response (red line).

Figure 4 shows the scalp distribution of power at the first and second harmonic, and the average SSVEP to the *CFS* and *noCFS* conditions. For all three *noCFS* we found extreme evidence the observed data are more likely under H_1_, δ > 0, than under H_0_, δ = 0: *neuFace_noCFS*, M = 0.35 µV, *SD* = 0.23, BF = 30,161, *fearFace_noCFS*, M = 0.31 µV, *SD* = 0.17, BF = 72,251, *object_noCFS*, M = 0.57 µV, *SD* = 0.31, BF = 290,318. In contrast, for each of the *CFS* conditions we found anecdotal to moderate evidence that the observed data are more likely under H_0_, δ = 0, than under H_1_, δ > 0: *neuFace_noCFS*, M = –0.02 µV, *SD* = 0.17, BF = 0.17, *fearFace_noCFS*, M = 0.02 µV, *SD* = 0.10, BF = 0.61, *object_noCFS*, M < .01 µV, *SD* = 0.06, BF = 0.24.

The results of the frequentist one-sample *t* tests were qualitatively the same as the Bayesian tests. A significant response was evoked by all of the *noCFS* conditions: *neuFace-noCFS*, *t*(19) = 6.97, *p* < .001, *d* = 1.56, *fearFace-noCFS*, *t*(18) = 7.65, *p* < .001, *d* = 1.75, *object-noCFS*, *t*(19) = 8.27, *p* < .001, *d* = 1.85. In contrast, the *CFS* conditions did not yield any significant effects, *p*s ≥ .16.

Paired-samples Bayesian *t* tests tested whether there was a difference in the SSVEP evoked by neutral or fearful faces in either the conscious or nonconscious conditions. We found anecdotal support of the null hypothesis of no difference in both the conscious, BF = 0.34, and nonconscious, BF = 0.36, conditions. As with the one-sample tests, the results of the analogous frequentist paired-samples *t* tests, *p*s > .35, were consistent with the Bayesian results. See Supplementary Material 1.2, Figure S2 for left hemisphere results.

### Combined study 1 and study 2 EEG results

To maximize SNR and thus detection sensitivity, we analyzed the combined *neuFace* SSVEP data from Study 1 and Study 2 resulting in a larger sample of *N* = 39. For the *neuFace*_*noCFS* condition, M = 0.34 µV, *SD* = 0.23, we found extreme evidence, BF = 6.699e+8, that the observed data are more likely under H_1_, δ > 0, than under H_0_, δ = 0,. In contrast, for the *neuFace* condition, M = −.01 µV, *SD* = 0.13, we found moderate evidence, BF = 0.13, that that observed the results are more likely (specifically, 7.81 times more likely) under H_0_, δ = 0, than under H_1_, δ > 0.

The results of the frequentist one-sample one-sided *t* tests were qualitatively the same as the Bayesian tests. A significant response was evoked by *neuFace-noCFS*, *t*(38) = 9.25, *p* < .001, *d* = 1.48, but not *neuFace*-*CFS* condition, *t*(38) = 0.43, *p* = .67. See Supplementary Material 1.3 for left hemisphere results.

### Study 3: Behavioral results

**Figure 5.**
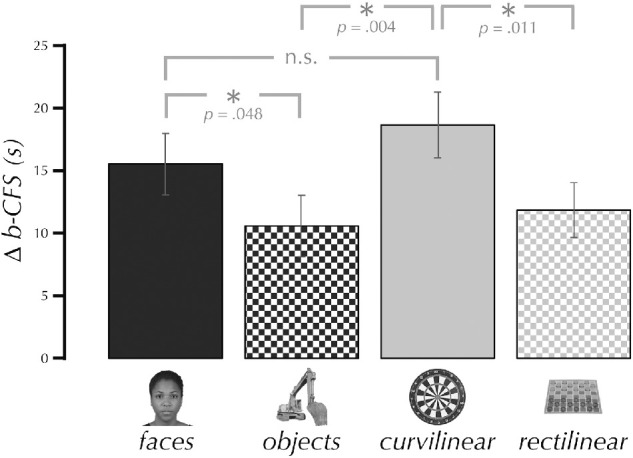
Breakthrough time advantage in Study 3. The bar graph shows the difference between the entire possible run duration and the actual average run duration. Thus, a larger number indicates a faster b-CFS. The data (*N* = 35) were analyzed with four planned one-way paired-samples *t* tests (see Methods).

Across all participants (*N* = 35), a one-way repeated-measures ANOVA showed that breakthrough time was significantly affected by condition, *F*(2.81, 95.57) = 3.60, *p* = .018 (Figure 5). One-way paired-samples *t* tests were used for four planned comparisons. The breakthrough time for *faces*, M = 15.56 s, *SD* = 14.52, was significantly faster than for *objects*, M = 10.60 s, *SD* = 14.53; *t*(34) = 1.71, *p* = .048, *d* = 0.29, but not *curvilinear* objects, M = 18.69 s, *SD* = 15.57; *p* = .910. The breakthrough time for *curvilinear* objects was significantly faster than for both *objects*, *t*(34) = 2.81, *p* = .004, *d* = 0.48, and *rectilinear* objects, M = 11.87 s, *SD* = 12.99; *t*(34) = 2.39, *p* = .011, *d* = 0.40.

Results of paired-samples Bayesian *t* tests were consistent with the planned comparisons reported above. We found anecdotal support for *faces* faster than for *objects*, BF = 1.29, and strong support supporting the null when testing *faces* faster than *curvilinear* objects, BF = 0.08. We found strong support for *curvilinear* objects was faster than *objects*, BF = 10.12, and moderate support for *curvilinear* objects faster than *rectilinear* objects, BF = 4.25.

## Discussion

We report two experiments that do not support the engagement of cortical face-selective regions during interocular suppression. A face-sensitive SSVEP to neutral faces (Studies 1 and 2) or fearful faces (Study 2) was observed only when participants were aware of the stimuli. In contrast, we observed evidence of selective nonconscious processing; faces broke through interocular suppression faster than objects. We followed up on these results with a third study in which we observed faster breakthrough time for curvilinear than rectilinear objects. Moreover, the breakthrough time for curvilinear objects did not differ from faces. We interpret these results as follows: 1) evidence that cortical face-selective regions are not engaged during face perception without awareness, 2) this is true for fearful, as well as neutral, faces, and 3) faster breakthrough times for faces is owed to the curvilinearity common to all faces rather than to high-level category membership.

### Evidence for the absence of an EEG correlate of nonconscious detection of neutral faces as indicated by Bayesian analysis

In Studies 1 and 2, we did not find an EEG response indicating face detection when faces were presented outside of conscious awareness. Is this finding simply another result added to a conflicted literature (for a recent review, see Axelrod et al., 2015) in which some have found magnetoencephalography and electroencephalography signals associated with nonconscious detection (Henson, Mouchlianitis, Matthews, & Kouider, 2008; Sterzer et al., 2009; Jiang et al., 2009; Suzuki & Noguchi, 2013), whereas others have not (Reiss & Hoffman, 2007; Harris et al., 2011; Navajas et al., 2013; Shafto & Pitts, 2015; Kume et al., 2016)? We believe not. Rather, the current results are a meaningful contribution to the literature given our unique combination of CFS and SSVEP, which addresses three possible limitations of the prior studies: 1) inconsistent power to detect face-selective EEG signals during nonconscious processing, 2) different blinding methods, and 3) variation in how each study operationalizes “awareness.”

The SSVEP approach has superior SNR to other EEG approaches like ERP (Norcia, Appelbaum, Ales, Cottereau, & Rossion, 2015), so the null effect reported here is less likely to be due to insufficient power. As noted in the Introduction, it has been argued that interocular rivalry paradigms are susceptible to partial awareness during CFS (Mudrik, Gelbard-Sagiv, Faivre, & Koch, 2013; Gayet, Van der Stigchel, & Paffen, 2014; Stein & Sterzer, 2014) that results in false positives. Here, we report evidence against face detection without awareness, despite using an approach that is ostensibly more likely to produce a positive result. Finally, because SSVEPs are based on the periodicity of the entire epoch, they are less sensitive to response biases that affect participants reporting of breakthrough. Perhaps most important, any concern that a response bias might lead to type I errors should be assuaged by the fact that we did not observe a positive SSVEP during periods which the participants did not report awareness.

### No evidence of nonconscious detection of fearful faces

Are emotionally relevant signals privileged relative to neutral signals? It has been proposed that affective signals are qualitatively different than neutral signals and processed via subcortical pathways (Tamietto & de Gelder, 2010; but see Pessoa & Adolphs, 2010) or, in the case of face processing, cortical pathways distinct from those that support identity processing (Haxby, Hoffman, & Gobbini, 2000; but see Calder & Young, 2005). Faivre, Berthet, and Kouider (2014) note that there is more consistent evidence for nonconscious processing of facial expression than there is for facial identity, and therefore that “The discrepancy between the processing of facial identity and facial expressions suggests that the latter may be processed along subcortical routes that are not fully disrupted by CFS” (p. 8). Support for an affective advantage comes primarily from functional magnetic resonance imaging and behavioral studies (for reviews, see Sterzer, Stein, Ludwig, Rothkirch, & Hesselmann, 2014; Axelrod et al., 2015; Diano, Celeghin, Bagnis, & Tamietto, 2017), although there are also a handful of EEG reports (Jiang et al., 2009; Jessen & Grossmann, 2014).

In the current work, we did not observe a behavioral affective advantage for fearful faces. Fearful and neutral faces broke through suppression faster than objects, but both frequentist and Bayesian analyses indicated no difference between them. That is, fearful faces did not capture conscious awareness faster than neutral faces. Similarly, neither fearful nor neutral faces evoked an SSVEP when presented nonconsciously. These results are consistent with several reports that suggest carefully controlling for potential methodological confounds causes any affective advantage to disappear (Straube, Dietrich, Mothes-Lasch, Mentzel, & Miltner, 2010; Pessoa & Adolphs, 2010; Hoffmann, Mothes-Lasch, Miltner, & Straube, 2015; Hedger, Adams, & Garner, 2015; Hedger, Gray, Garner, & Adams, 2016). Schlossmacher, Junghöfer, Straube, and Bruchmann (2017) found that modulation of face-sensitive ERPs (e.g., N170) observed during conscious perception were not observed during interocular rivalry with CFS. Finally, the authors of a recent meta-analysis of behavioral experiments conclude that “uncritical acceptance of the standard hypothesis, which states that threat stimuli can be identified and prioritized without awareness, is premature” (Hedger et al., 2016, p. 961). But, notably, they report that fearful faces were the only threat stimulus that consistently showed evidence of a nonconscious advantage in b-CFS paradigms. Therefore, we must entertain the possibility that there is an affective advantage in nonconscious processing with a source that is not readily detectable with EEG.

### Is there a subcortical effect?

A limitation of EEG, and thus the current work, is that potentials generated in subcortical structures will have a lower SNR owing to their increased distance from recording sites on the scalp. For some structures, such as the amygdala, this is exacerbated by a spatial organization of neurons that results in local volume currents cancelling each other out rather than summating into a field large enough to be detected on the scalp (Silva, 2018). The current pattern of results—faster breakthrough times despite the absence an of EEG signature—would be consistent with faces being processed nonconsciously by subcortical systems and would explain the behavioral advantage without concomitant SSVEP. This was our initial conclusion after seeing the results of Study 1. However, the full pattern of results across all three studies makes this unlikely for at least two reasons.

First, if a subcortical pathway existed for fast processing and thus attentional orienting, one would reasonably assume that this system would engage the relevant cortical systems that are specialized for processing the to-be-attended stimuli (Brooks et al., 2012). In contrast, we found no indication of cortical engagement, although we note that there are studies which have found evidence of amygdala activation without concomitant cortical activation (see de Gelder, van Honk, & Tamietto, 2011). Second, and perhaps more important, we did not observe a faster breakthrough time for fearful faces compared with neutral faces. If anything, the breakthrough time for fearful faces was slower (although this was a small and insignificant difference). So, on the one hand, the current data cannot rule out subcortical nonconscious processing of fearful faces. On the other hand, if such processing occurs, it does so without engaging cortical face processing systems and without conferring an observable behavioral advantage to fearful faces.

### The effect of mid-level visual features on b-CFS

At first blush, the EEG and behavioral results of Studies 1 and 2 seem to be incompatible. We observed a significantly faster breakthrough time for faces than objects, but no face-selective SSVEP. We believe the results of Study 3—faster breakthrough times for curvilinear than for rectilinear objects—elucidate the nature of this contradiction. Specifically, we interpret these results as evidence that the behavioral advantage for face processing is owed to the curvilinearity of faces rather than their high-level category membership. This interpretation is consistent with a growing literature that focuses on the importance of mid-level feature processing in the visual system.

Perhaps most relevant to the current work is a recent study by Moors, Wagemans, and de-Wit (2016) in which b-CFS was investigated as a function of curvature relative to fixation. Participants viewed the left or right half of a face in either an upright or inverted orientation, presented to the left or right of fixation. Thus, faces were either presented with natural convex, or unnatural concave curvature relative to fixation. They found that curvature relative to fixation played an important role in faster breakthrough such that natural convexity was faster than concavity. This finding is consistent with prior work that found a preference for convex contours in area V4 of the macaque (Pasupathy & Connor, 1999). In the current studies, all curvilinear images (faces and objects) were convex relative to fixation, so the data cannot speak to the importance of convexity versus concavity, but do support priority for curvilinear over rectilinear contours.

We presented all stimuli roughly centered at fixation, thus resulting in processing occurring primarily in regions of visual cortex with foveal and parafoveal receptive fields. This condition might contribute to the observed nonconscious preference for curvilinear shapes. In macaque visual cortex there is a correlation between contour and eccentricity, such that curvilinear contours are preferred in the central visual field, whereas rectilinear contours are preferred in the periphery (Srihasam, Vincent, & Livingstone, 2014). This observed relationship is particularly strong in early visual cortex, but a general preference for curvature has been observed to increase from lower to higher visual processing areas (Wilkinson et al., 2000; Ponce, Hartmann, & Livingstone, 2017) and might contribute to the organization of high-level visual cortex (Nasr, Echavarria, & Tootell, 2014; Srihasam et al., 2014; Andrews, Watson, Rice, & Hartley, 2015; Long, Yu, & Konkle, 2018).

Human face selective regions are particularly sensitive to curvilinearity (Caldara et al., 2006). Indeed, prosopagnosia (aka face blindness) seems to selectively impair the processing of curved edges and shapes (Kosslyn, Hamilton, & Bernstein, 1995). Similarly, a network of curvature-sensitive regions in the macaque brain is adjacent to face-sensitive regions, suggesting a possible functional relationship (Yue, Pourladian, Tootell, & Ungerleider, 2014). There is also an intriguing relationship between curvilinearity and animacy, such that behavioral categorization largely depends on the amount of curvilinearity present in the image with images of animate things being more curvilinear than images of inanimate things (Long, Störmer, & Alvarez, 2017; Zachariou, Del Giacco, Ungerleider, & Yue, 2018; but also see Proklova, Kaiser, & Peelen, 2016). Furthermore, a recent ERP study found evidence that animals and non-animals were distinguished nonconsciously (Zhu, Drewes, Peatfield, & Melcher, 2016).

Do mid-level features, particularly curvilinearity, account for observed differences in b-CFS paradigms? Our results are consistent with prior reports that would suggest so. Such features have been shown to drive the nonconscious processing of face identity (Gelbard-Sagiv, Faivre, Mudrik, & Koch, 2016), expression (Hedger et al., 2015), and dominance (Stein, Awad, Gayet, & Peelen, 2018; Gayet et al., 2014), although it is yet unclear whether these features are themselves being processed nonconsciously (Pitts, Martínez, & Hillyard, 2012), or if the effect is due to partial awareness (Gelbard-Sagiv et al., 2016).

### Two-Threshold model

The contribution of mid-level features discussed elsewhere in this article might account for many of the studies that have reported nonconscious processing of several different dimensions of face perception (for a review, see Axelrod et al., 2015), but others are less easily explained. For example, Gobbini, Gors, Halchenko, Hughes, and Cipolli (2013) report that faces oriented directly toward the viewer breakthrough faster than faces oriented slightly away. In this case, both conditions have near-identical curvilinearity and, importantly, convexity relative to fixation. What might drive this effect if not mid-level features?

One intriguing possibility is that nonconscious processing is not an all-or-none phenomenon, but rather can be considered a process of degree. This “two-threshold model of nonconscious processing” (Schlossmacher et al., 2017) posits that some features might only be processed when in the shallow depths of unconsciousness (Peremen & Lamy, 2014; Sterzer et al., 2014). In the context of this model, mid-level features might push faces from the depths toward the waterline of consciousness, at which point they are susceptible to privileged processing that ultimately causes a faster breakthrough. On the one hand, our results can be interpreted as being broadly consistent with such a model. In contrast, we did not observe an advantage of fearful faces compared to neutral faces, or neutral faces compared with curvilinear objects. In other words, we did not observe an additive benefit of high-level category membership (face vs. object) beyond what could be explained by mid-level features (curvilinear vs. rectilinear).

### Limitations

We have addressed several limitations of the current work in the prior discussion. Here we briefly address three more. First, we used low opacity images (see Methods) to extend suppression time. It is possible that this choice accounts for our inability to detect an EEG response, but this seems unlikely because we did observe a behavioral effect despite the low opacity. It should also be noted that the low opacity images evoked a sufficient signal in the *noCFS* conditions. Second, faces are a substantially more homogenous set of stimuli than are objects. It is possible that the repetition of homogenous oddballs facilitated a faster breakthrough time. We think this is unlikely given the design and the results of Study 3. In that study, we observed a faster breakthrough time for curvilinear objects than for rectilinear objects, despite there being no appreciable difference in the homogeneity of the seven exemplars within each condition. Third, it is possible that the observed curvilinearity effect was owed to contrast with the rectilinear continuous flash stimuli. We cannot exclude this possibility, because these studies do not include a version in which the flash suppression stimuli are curvilinear. The substantial evidence for the importance of curvilinearity in both low-level and category-selective regions of the visual system leads us to conclude that this explanation is unlikely. Further, both faces and curvilinear objects were suppressed with rectilinear CFS stimuli, so this could not account for the important observation that the breakthrough times did not differ for faces and curvilinear objects.

## Conclusions

The results of these studies suggest that cortical face-selective regions do not engage in nonconscious face processing. Moreover, the observed advantage faces have over non-faces in breaking through flash suppression is likely due to their curvilinearity, rather than their high-level category membership. In the current series of studies, we were unable to find EEG evidence in support of the notion that faces are processed without benefit of conscious awareness. Paradoxically, we did observe a faster breakthrough time into conscious awareness for faces than for objects. A faster b-CFS is commonly interpreted to indicate nonconscious processing (Jiang et al., 2007). In a follow-up study, we found evidence that the mid-level visual features of a face—specifically, their curvilinearity—account for the faster breakthrough time, rather than their high-level category membership, and therefore are why we see no EEG signature of face perception.

## Supplementary Material

Supplement 1

Supplement 2

Supplement 3
