# Supplementary material for: Faces under continuous flash suppression capture attention faster than objects, but without a face-evoked steady-state visual potential: Is curvilinearity responsible for the behavioral effect?
Source: J Vis. 2020 Jun 17;20(6):14. doi: 10.1167/jov.20.6.14 (PMC7416886; doi:10.1167/jov.20.6.14)

Figure 2. Study 2 SSVEP: These 3-D (larger) and 2-D (smaller inset) scalp maps display the distribution of normalized power at the first harmonic (aka fundamental) of the oddball presentation frequency (a). During *noCFS* there was no interocular suppression and the participants were therefore consciously aware of all presented stimuli. During *CFS* there was interocular suppression and the participants were therefore unaware of the stimuli of interest presented to the 'suppressed' eye. The bean plots (b) display the average amplitude of the response (µV) combined across the first and second harmonics and across three electrodes of interest: P7, P9, and PO9 for each participant. For each condition, the plot displays the individual participant results (black lines), the distribution density of the results (mirrored across the vertical axis), and the mean response (red line).

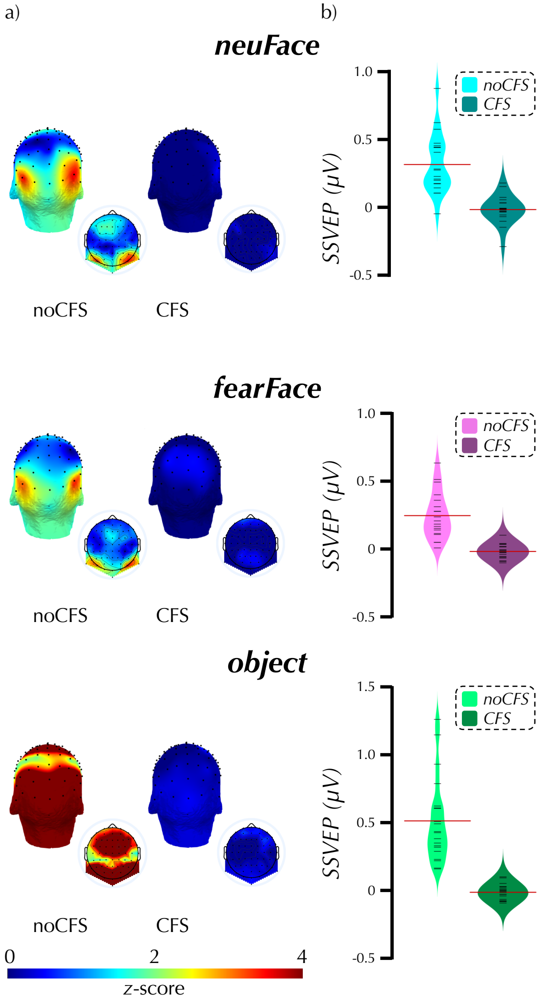

Supplement: Supplement 2 [file jovi-20-6-14_s002.pdf]
