# Supplementary material for: Faces under continuous flash suppression capture attention faster than objects, but without a face-evoked steady-state visual potential: Is curvilinearity responsible for the behavioral effect?
Source: J Vis. 2020 Jun 17;20(6):14. doi: 10.1167/jov.20.6.14 (PMC7416886; doi:10.1167/jov.20.6.14)
Supplement: Supplement 3 [file jovi-20-6-14_s003.docx]

# EEG Results from Left Hemisphere ROI (PO7, P7, P9)

## Study 1:

Figure 1 shows the scalp distribution of power at the first and second harmonic, and the average SSVEP to the *CFS* and *noCFS* conditions. For the *neuFace*-*noCFS* condition (M = .29 µV, SD = .17) we found extreme evidence (BF = 78,457) that the observed data are more likely under H_1_ (δ > 0) than under H_0_ (δ = 0). For the *neuFace* condition (M = .05 µV, SD = .08) we found moderate evidence (BF = 5.49) that the results are more likely are more likely under H_1_ (δ > 0) than under H_0_ (δ = 0).

The results of the frequentist one-sample one-sided *t*-tests were qualitatively the same as the Bayesian tests. A significant response was evoked by both the *neuFace-noCFS* (*t*(18) = 7.70, *p* < .001, *d* = 1.77), and *neuFace-CFS* condition (*t*(18) = 2.52, *p* = .011, *d* = 0.58).

At first blush, these results run counter to those in the right hemisphere and suggest nonconscious processing of faces. However, as reported below, this does not replicate in Study 2 neutral or fearful faces. Further, the effect does not survive when combining the samples of Study 1 and Study 2.

## Study 2

Figure 2 shows the scalp distribution of power at the first and second harmonic, and the average SSVEP to the *CFS* and *noCFS* conditions. For all three *noCFS* we found extreme evidence the observed data are more likely under H_1_ (δ > 0) than under H_0_ (δ = 0): *neuFace_noCFS* (M = .31 µV, SD = .22, BF = 11,706), *fearFace_noCFS* (M = .26 µV, SD = .17, BF = 13,218), *object_noCFS* (M = .53 µV, SD = .31, BF = 108,253). In contrast, for each of the *CFS* conditions we found anecdotal to moderate evidence that the observed data are more likely under H_0_ (δ = 0) than under H_1_ (δ > 0): *neuFace_noCFS* (M = -.02 µV, SD = .10, BF = .15), *fearFace_noCFS* (M = .026 µV, SD = .17, BF = .20), *object_noCFS* (M < .001 µV, SD = .06, BF = .40).

The results of the frequentist one-sample *t*-tests were qualitatively the same as the Bayesian tests. A significant response was evoked by all of the *noCFS* conditions: *neuFace-noCFS* (*t*(19) = 6.47, *p* < .001, *d* = 1.45), *fearFace-noCFS* (*t*(18) = 6.66, *p* < .001, *d* = 1.53), *object-noCFS* (*t*(19) = 7.69, *p* < .001, *d* = 1.72). In contrast, the *CFS* conditions did not yield any significant effects (*p*s ≥ .27).

Paired-samples Bayesian *t*-tests tested whether there was a difference in the SSVEP evoked by neutral or fearful faces in either the conscious or nonconscious conditions. We found anecdotal support of the null hypothesis of no difference in both the conscious (BF = .34) and nonconscious (BF = .27) conditions. As with the one-sample tests, the results of the analogous frequentist paired-samples *t*-tests (*p*s > .37) were consistent with the Bayesian results.

## Combined Study 1 and Study 2 EEG results

In order to maximize SNR and thus detection sensitivity, we analyzed the combined *neuFace* SSVEP data from Study 1 and Study 2 resulting in a larger sample of N = 39. For the *neuFace*_*noCFS* condition (M = .30 µV, SD = .19) we found extreme evidence (BF = 3.937e+9) that the observed data are more likely under H_1_ (δ > 0) than under H_0_ (δ = 0). In contrast, for the *neuFace* condition (M = .02 µV, SD = .10) we found anecdotal evidence (BF = .42) that that observed the results are more likely under H_0_ (δ = 0) than under H_1_ (δ > 0).

The results of the frequentist one-sample one-sided *t*-tests were qualitatively the same as the Bayesian tests. A significant response was evoked by *neuFace-noCFS* (*t*(38) = 9.25, *p* < .001, *d* = 1.59), but not *neuFace*-*CFS* condition (*t*(38) = .96, *p* = .172).
